# Recombinant norovirus-specific scFv inhibit virus-like particle binding to cellular ligands

**DOI:** 10.1186/1743-422X-5-21

**Published:** 2008-01-31

**Authors:** Khalil Ettayebi, Michele E Hardy

**Affiliations:** 1 Veterinary Molecular Biology, Montana State University, Bozeman, MT 59717, USA

## Abstract

**Background:**

Noroviruses cause epidemic outbreaks of gastrointestinal illness in all age-groups. The rapid onset and ease of person-to-person transmission suggest that inhibitors of the initial steps of virus binding to susceptible cells have value in limiting spread and outbreak persistence. We previously generated a monoclonal antibody (mAb) 54.6 that blocks binding of recombinant norovirus-like particles (VLP) to Caco-2 intestinal cells and inhibits VLP-mediated hemagglutination. In this study, we engineered the antigen binding domains of mAb 54.6 into a single chain variable fragment (scFv) and tested whether these scFv could function as cell binding inhibitors, similar to the parent mAb.

**Results:**

The scFv_54.6 _construct was engineered to encode the light (V_L_) and heavy (V_H_) variable domains of mAb 54.6 separated by a flexible peptide linker, and this recombinant protein was expressed in *Pichia pastoris*. Purified scFv_54.6 _recognized native VLPs by immunoblot, inhibited VLP-mediated hemagglutination, and blocked VLP binding to H carbohydrate antigen expressed on the surface of a CHO cell line stably transfected to express α 1,2-fucosyltransferase.

**Conclusion:**

scFv_54.6 _retained the functional properties of the parent mAb with respect to inhibiting norovirus particle interactions with cells. With further engineering into a form deliverable to the gut mucosa, norovirus neutralizing antibodies represent a prophylactic strategy that would be valuable in outbreak settings.

## Background

Noroviruses are non-enveloped positive strand RNA viruses that cause foodborne illness worldwide [1]. They are classified as NIAID Category B priority pathogens because they are easily transmitted person-to-person and can cause persistent epidemics. Outbreaks generally occur in semi-closed community settings including day care centers, retirement facilities and nursing homes, hospitals, schools, and military training and operations facilities. Large outbreaks on commercial cruise-liners have been well publicized, and such outbreaks illustrate the rapid onset epidemic potential of noroviruses and a need for intervention measures that do not depend on pre-existing immunity. Recent data suggest the number of outbreaks attributable to noroviruses may be increasing [2].

The norovirus genome is a 7.7 kilobase RNA comprised of three open reading frames (ORF) [reviewed in [3]]. ORF1 codes for the nonstructural proteins that are processed co- and post-translationally by a single viral protease. ORF2 and ORF3 encode structural proteins VP1 and VP2, respectively, and form the icosahedral capsid. Ninety dimers of VP1 assemble into virus-like particles (VLPs) when expressed in insect cells infected with a recombinant baculovirus [4]. VP1 folds into two major domains termed the shell (S) and protruding (P) domains [5,6]. The S domain consists of the N -terminal 280 amino acids and forms the icosahedron. The P domain is divided into sub-domains P1 and P2 that participate in dimeric contacts that increase the stability of the capsid. The P2 domain is an insertion in the P1 domain and contains a hypervariable region implicated in receptor binding and immune reactivity, as well as in interactions with histoblood group antigens associated with susceptibility to norovirus infections [7-11].

Therapeutic antibodies have been used successfully in treatment regimens for diseases including cancer and rheumatoid arthritis, for transplant rejection, and against respiratory syncytial virus infections in children [reviewed in [12]]. Technological advances that include humanization to avoid undesirable immunogenicity, and improvements in stability and pharmacokinetics are strategies employed to improve the clinical utility of antibodies. Foremost among such strategies is the reduction of antigen binding domains to minimal fragments that retain reactivity with the targeted antigens [13]. Single chain variable fragments (scFv) are ~27 kDa recombinant proteins that consist of the light (V_L_) and heavy (V_H_) chain variable regions of a monoclonal antibody (mAb) expressed in a single construct where they are separated by a flexible peptide linker [14]. Intramolecular folding of the recombinant protein results in reconstitution of the antigen binding domain. These small proteins are relatively easily produced in high yield in recombinant bacterial or yeast expression systems [15-17]. Further manipulation and expression strategies have yielded forms of the scFv monomer where valency is increased by assembly of multimeric forms termed diabodies, triabodies and tetrabodies [13]. These multimers have been shown to be more stable and can be engineered to recognize more than one antigenic target [18,19].

We generated mAb to norovirus VLPs to characterize domains of VP1 that function in virus binding to cellular receptors [20]. One mAb (mAb 54.6) to the genogroup I reference strain Norwalk (NV) blocks binding of recombinant VLPs to CaCo-2 intestinal cells and inhibits VLP-mediated hemagglutination. In the current study, we engineered sequences encoding mAb 54.6 into an scFv to determine whether functional activity was retained in the isolated antigen binding domain. The data presented show the scFv from mAb 54.6 (scFv_54.6_) was expressed successfully in *Pichia pastoris *and retained the antigen binding and functional activity of the parent mAb. Engineered antibody fragments that block norovirus binding to cells have potential as an on-site prophylactic strategy to prevent virus spread and contain epidemics.

## Results

### V_L _and V_H _domains of mAb 54.6 and design of scFv_54.6_

**Figure 1:**
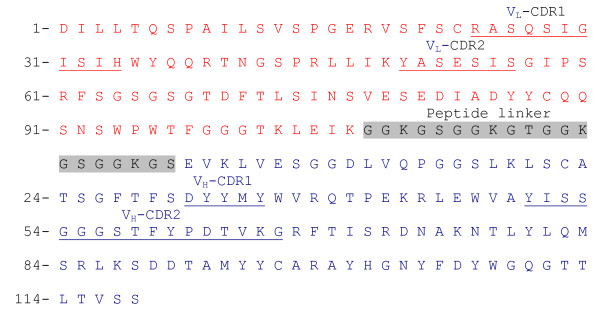
Amino acid sequence and domain organization of scFv_54.6_. V_L _(red) and V_H _(blue) chains are shown as a single sequence joined together with a 20 amino acid linker (shaded). Complementarity determining regions (CDRs) of V_L _and V_H _are underlined. Amino acid numbering is according to Kabat [43].

Anti-rNV mAb 54.6 recognizes non-denatured VP1, inhibits VLP-mediated hemagglutination, and blocks VLP binding to CaCo-2 cells. To determine whether functional activity of the mAb could be reduced to a smaller antigen binding domain, sequences encoding the V_L _and V_H _genes of mAb 54.6 were cloned from the hybridoma cells (Figure 1). A database search for homologies to known murine V genes was performed with the IgBLAST protocol. The V_L _domain of mAb 54.6 is 98.9% identical to V_L _genes in the Vκ-23-48 family [21], with the exception of a substitution of isoleucine for threonine at position 31 in the Vκ-CDR1. The V_H _domain was amplified in a single form highly homologous to the V_H_7183 gene family [22] and is 96% identical to the V_H _50.1 gene. V_H _genes derived from the V_H_7183 family are preferentially used by autoantibodies of various specificities [23]. However, some antibodies specific for foreign antigens such as galactan and the A/PR8/34 strain of influenza virus hemagglutinin also are encoded by V_H _genes of this family [24,25].

The domain organization of the 245 amino acid scFv_54.6 _consists of the V_L _domain at the N terminus separated from the V_H _domain at the C terminus by a peptide linker comprised of the sequence GGKGSGGKGTGGKGSGGKGS (Figure 1). The linker composition is a modification of the peptide linker previously described [26]. C-terminal myc and histidine tags are expressed in frame with scFv_54.6 _to allow detection of recombinant protein by immunoblot and for downstream purification procedures.

### Production of scFv_54.6 _in *P. pastoris *and reactivity with rNV VP1

**Figure 2:**
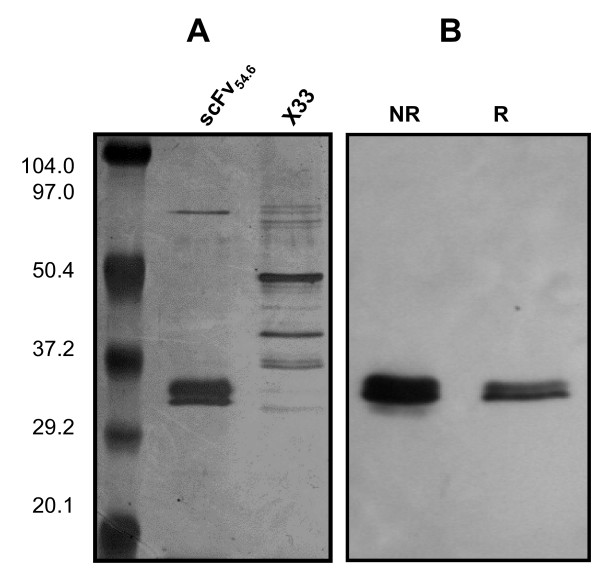
Expression of scFv_54.6 _in *P. pastoris*. (A) SDS-PAGE analysis of affinity chromatography purified scFv_54.6 _after 72 hrs induction. (B) Purified scFv_54.6 _electrophoresed under non-reducing (NR) and reducing (R) conditions was probed with anti-c-myc mAb and HRP-conjugated goat anti-mouse IgG.

The pPICZαA vector contains the methanol-inducible AOX1 promoter for high level expression in *P. pastoris *and the *α*-factor signal sequence from *Saccharomyces cerevisiae *for secretion of recombinant protein into the culture supernatant [27,28]. During secretion, the signal peptide is cleaved by the *P. pastoris *protease KEX2 (kexin), releasing soluble scFv_54.6 _into the medium. Typical yields following induction of expression with methanol, purification and concentration, were approximately one mg of purified scFv_54.6 _per liter of suspension culture. scFv_54.6 _has a calculated molecular mass of approximately 29 kDa. Three closely migrating protein bands were detected by silver stain (Figure 2A). The top two bands strongly reacted with the anti c-myc mAb under both reducing and non-reducing conditions (Figure 2B). The exact composition of the two bands is not clear, but they likely are products of slightly different KEX protease cleavage sites at the N terminus of the protein, because the myc epitope tag recognized by the antibody resides at the C terminus. In support of this interpretation, N-terminal amino acid sequence analysis of three forms of recombinant protein rhIFN-λ1 showed proteolytic processing adjacent to and outside of the KEX2 cleavage site [29].

**Figure 3:**
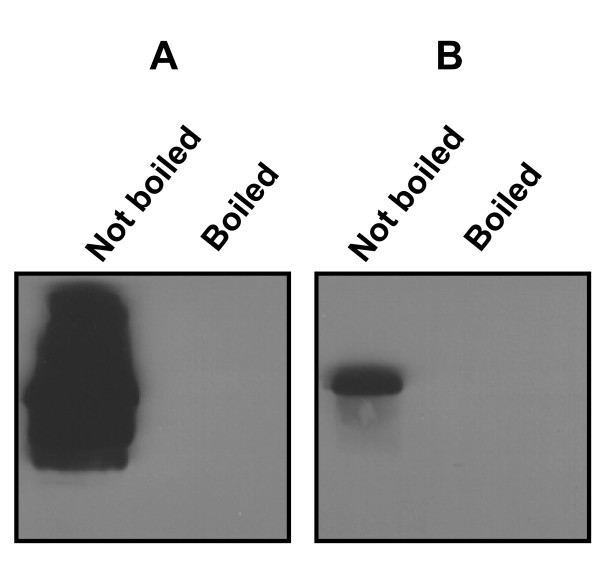
Western blot analysis of rNV VP1 probed with (A) mAb 54.6 and (B) scFv_54.6 _under non-denaturing (left panel) and denaturing (right panel) conditions.

Recognition of rNV VP1 by mAb 54.6 is conformation dependent because this antibody reacts in immunoblots only when VP1 has not been denatured by boiling in SDS and β-mercaptoethanol [20]. scFv_54.6 _was tested for the ability to bind rNV VP1 in immunoblots to determine whether the fragment retained reactivity of the parent mAb. Immunoblots were probed with scFv_54.6 _and binding to rNV VP1 was detected with anti-c-*myc *mAb and goat anti-mouse secondary antibody. Similar to mAb 54.6, scFv_54.6 _recognized VP1 only under non-denaturing, non-reducing conditions (Figure 3). Neither the mAb 54.6 nor scFv_54.6 _recognized denatured VP1.

### scFv_54.6 _blocks rNV VLP-mediated hemagglutination

**Figure 4:**
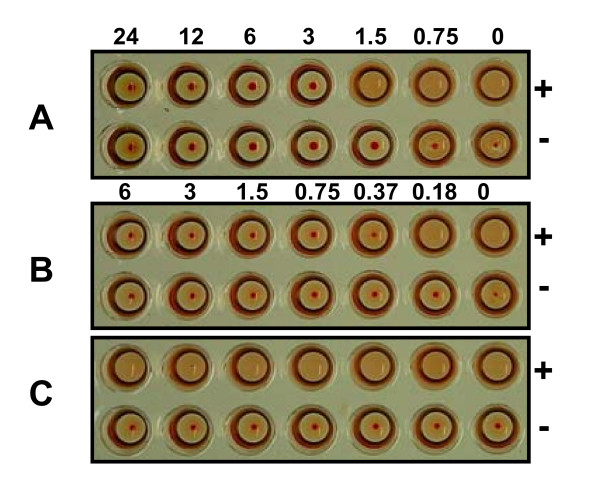
Dose-dependent hemagglutination inhibition by recombinant scFv_54.6_. Numbers above the wells indicate the amount of antibody in the reactions in μg. (A) scFv_54.6 _(B) mAb 54.6 (C) PBS-H and rbc only. (+) indicates presence of rNV particles, (-) indicates absence of rNV particles.

Norovirus VLPs agglutinate red blood cells in a type-specific manner [30] and mAb 54.6 inhibits this activity for rNV VLPs [20]. To determine whether scFv_54.6 _inhibited hemagglutination, rNV VLPs were mixed with type O rbc in the presence or absence of scFv_54.6 _or mAb 54.6. Figure 4A shows that scFv_54.6 _successfully blocked rNV VLP-mediated hemagglutination in a dose-dependent manner. The lowest inhibitory concentration was 3.0 μg and 0.375 μg of scFv_54.6 _and the parent mAb, respectively (Figures 4A and 4B), yielding an HI titer for scFv_54.6 _eight-fold lower than that of mAb 54.6. While it is possible that part of the difference in HI titer can be attributed to slight differences in concentration, it is likely that the lower HI titer of scFv_54.6 _reflects the monovalent binding activity of the fragment.

### scFv_54.6 _blocks binding of rNV VLPs to CHO-FTB_KE _cells

**Figure 5:**
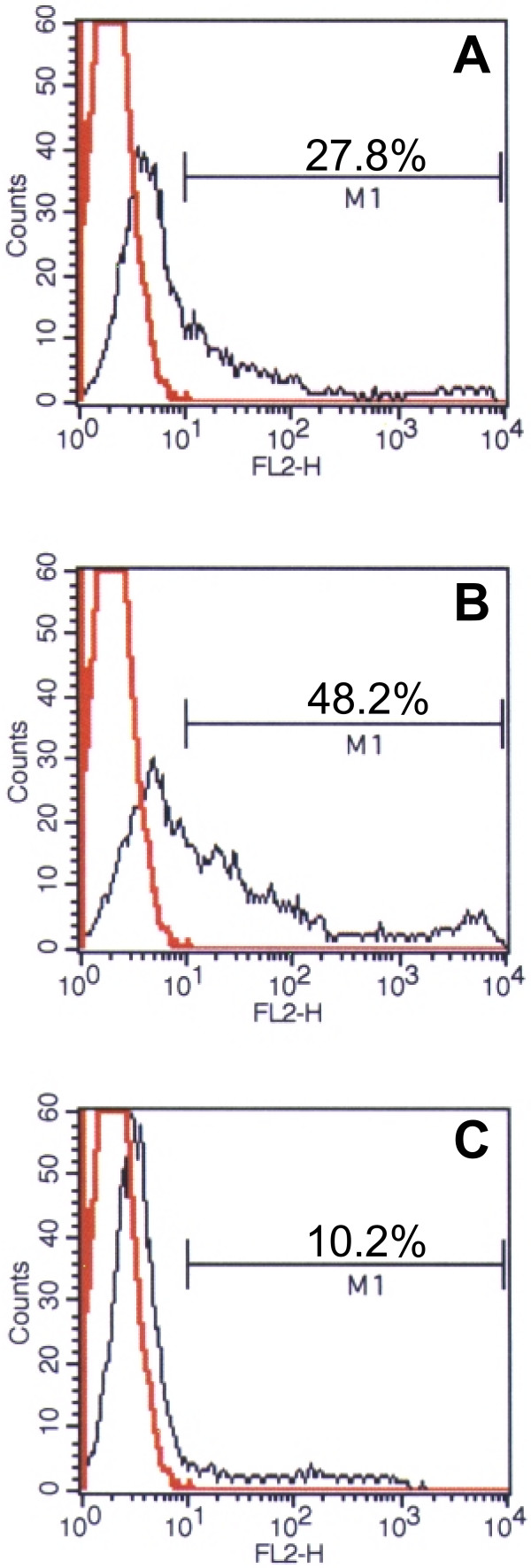
scFv_54.6 _blocks the binding of rNV VLPs to CHO-FTB_KE _cells. rNV VLPs were incubated with CHO-FTB_KE _cells in the presence or absence of scFv_54.6 _and sorted by flow cytometry (A) VLP binding detected by incubation with scFv_54.6 _followed by anti-c-myc mAb and PE-conjugated goat anti-mouse antibody. (B) VLP binding detected by incubation with anti rNV MAb 72.1 PE-conjugated goat anti-mouse and C) scFv_54.6 _were pre-incubated with VLPs for one hour prior to addition to the cells. Bound VLPs were detected by MAb 72.1.

CHO cells do not express ABH histoblood group antigens, but CHO cells stably transfected with the rat FTB gene encoding α 1,2-FT confers the ability to bind rNV VLPs to these cells [31]. scFv_54.6 _was tested for the ability to block binding of rNV VLPs to CHO-FTB_KE _cells. In the absence of antibody, 48.2% of cells bound VLPs (Figure 5). When VLPs were pre-incubated with scFv_54.6_, the percentage of positive cells was reduced to 10.2%. No reduction in binding was observed with samples from non-expressing X33 culture subjected to the same purification procedure as the scFv_54.6 _(see Figure 2A). These results suggest scFv_54.6 _is able to block binding of rNV VLPs to H type antigen on the cell surface.

## Discussion

Immunity to noroviruses is complex and the correlates of protection from re-infection are unclear. Short-term immunity can be induced by prior infection, but long-lasting immunity apparently is more difficult to achieve. Gastroenteritis caused by norovirus generally is a self-limiting disease. However, the ease in which noroviruses are transferred by person-to-person spread suggest inhibitors designed to interfere with the initial steps of virus infection could reduce the duration of illness, shedding of infectious virus, and the number of susceptible individuals in a population. This issue is particularly relevant in semi-closed communities including nursing homes and daycare centers, where age may affect the efficacy of vaccines. We envision that inhibitors will be easily administered on-site as necessary and will by design, be independent of an adaptive immune response.

*P. pastoris *was chosen for scFv_54.6 _expression because of reported high protein yield, low levels of glycosylation, simplicity of the culture medium and protein solubility. Numerous scFvs have been produced in *P. pastoris *with variable yields ranging from 0.4 to 20 mg/L [26,32,33]. We have shown that scFv_54.6 _expressed in *P. pastoris *at yields of ~1.0 mg/L retain the inhibitory functions of the parent mAb, suggesting humanized derivatives of these fragments could prove useful for development of anti-norovirus prophylactics.

Antibodies have received significant interest as biopharmaceuticals. At least 18 mAb are in use in humans and > 100 are in clinical trials as therapeutics for treatment of cancer and chronic inflammatory diseases, and for prevention of transplant organ rejection [12]. Likewise, engineered smaller domains consisting of the functional antigen binding domains are viable alternatives to the costs associated with humanization and production of mAb in mammalian cell culture systems [13]. Monovalent scFv are easily produced in high yield in recombinant bacterial or yeast expression systems, and when secreted into the medium, are readily purified to homogeneity by scalable purification procedures. scFv also can be designed to be bispecific, or engineered to preferentially form multimers including diabodies, triabodies, and tetrabodies that increase valency [13]. These multivalent properties with consequent increased avidity are particularly relevant for prophylaxis of viral diseases because, similar to other cellular receptor-ligand interactions, viral attachment proteins engage multiple copies of receptors on the cell surface.

Several examples of antibody fragments capable of neutralizing virus infection both in vitro and in vivo have been reported. For example, fragments selected from a human scFv library for reactivity with the West Nile virus envelope protein protected mice against lethal virus challenge when administered both prior to or shortly after infection [34]. Recombinant virus-specific scFv efficiently neutralized human papillomavirus infection in culture [35]. Affinity-selected scFv against hepatitis B virus also neutralized infection in vitro [36], and purified scFv that recognize intercellular adhesion molecule ICAM-1 effectively blocked transmission of HIV across an epithelial cell monolayer both in vitro and in a small animal model [37]. Together, these data illustrate the utility of scFv as a viable way to provide passive immunity to viral infections.

Our studies were approached with the idea that engineered antibody fragments could provide a rapidly deliverable substance for protection against norovirus infection where the potential for epidemics is heightened in semi-closed community settings. However, successful delivery of soluble proteins, including scFv, to the gut is unlikely because of potential instability from exposure to gut proteases. Recently, pathogen-specific scFv expressed on the surface of probiotic lactobacilli provided protection in small animal models of enteric viral infection [38]. This or a similar system that is amenable to oral administration could provide scaffolding for stable presentation of norovirus neutralizing scFv to the gut.

The antigenic specificity in the norovirus capsid lies primarily in the hypervariable P2 domain of VP1, and ideally, antibody fragments would be reactive with multiple strains. Genogroup II.4 strains currently are the predominant strains circulating worldwide, but others also have been reported [2,39-42]. In the absence of broadly cross-reactive antibodies, the ability to manipulate scFv fragments into bispecific molecules suggests these neutralizing fragments can be engineered to cover a combination of antigenic types within a single delivery system. Construction of such bispecific fragments is underway.

## Material and methods

### Cloning of variable domain genes

Total RNA was isolated from hybridoma 54.6 [20] with the RNeasy Mini kit (Qiagen). Reverse transcription (RT) was conducted with 5 μg of total RNA using M-MLV RT (Invitrogen) and 3' oligonucleotides MVK-R and MVH-R (Table 1) for first-strand synthesis of cDNA corresponding to the variable regions of the light and heavy chains, V_L _and V_H_, respectively. Two μl of the cDNA reaction were used in PCR reactions conducted with degenerate 5' oligonucleotides for leader sequences (Table 1) and the 3' oligonucleotides used in the RT reaction. A single PCR amplicon obtained in each reaction was cloned into the pCR2.1 TOPO vector (Invitrogen), and sequenced using M13 reverse and M13 forward (-20) primers. The V_L _and V_H _sequences then were amplified from the pTOPO clones using the oligonucleotides described in Table 2. Both V_L _and V_H _PCR products were digested with *Kpn I *and ligated with T4 DNA ligase. The ligation products were used as templates to amplify the joined V_L _and V_H _regions separated by a 60 bp linker. The resulting PCR products then were cloned into the *Pichia pastoris *expression vector pPICZαA (Invitrogen) utilizing the *EcoR I *and *Xba I *restriction sites to yield pPICZαA-scFv_54.6_. Colonies were selected on zeocin-containing low salt agar plates containing 1% tryptone, 0.5% yeast extract, 0.5% sodium chloride and 25 μg/ml zeocin.

**Table 1:** Degenerate oligonucleotides for PCR amplification of V_L _and V_H _of mAb 54.6

MVH-R	5'- GAC HGA TGG GGS TGT YGT GCT AGC TGN RGA GAC DGT GA -3'
MVK-R	5'- GGA TAC AGT TGG TGC AGT CGA CTT ACG TTT KAT TTC CAR CTT -3'
VK1-F	5'- GGG GAT ATC CAC CAT GGA GAC AGA CAC ACT CCT GCT AT -3'
VK2-F	5'- GGG GAT ATC CAC CAT GGA TTT TCA AGT GCA GAT TTT CAG -3'
VK3-F	5'- GGG GAT ATC CAC CAT GGA GWC ACA KWC TCA GGT CTT TRT A -3'
VK4-F	5'- GGG GAT ATC CAC CAT GKC CCC WRC TCA GYT YCT KGT -3'
VK5-F	5'- GGG GAT ATC CAC CAT GAA GTT GCC TGT TAG GCT GTT G -3'

VH1-F	5'- GGG GAT ATC CAC CAT GGR ATG SAG CTG KGT MAT SCT CTT -3'
VH2-F	5'- GGG GAT ATC CAC CAT GRA CTT CGG GYT GAG CTK GGT TTT -3'
VH3-F	5'- GGG GAT ATC CAC CAT GGC TGT CTT GGG GCT GCT CTT CT -3'
VH4-F	5'- GGG GAT ATC CAC CAT GAT RGT GTT RAG TCT TYT GTR CCT G -3'

**Table 2:** Primers for cloning V_L _and V_H _of mAb 54.6 into *P. pastoris *expression vector pPICZαA

VKF-54.6	5'- CAC GAA TTC GAC ATC TTG CTG ACT CAG TCT CCA GCC -3'
VKLR-54.6	5'- CTA CGG TAC CCT TAC CTC CAG ATC CCT TAC CTC CTT TGA TTT CCA ACT TGG TGC C -3'
VHLF-54.6	5'- CAC GGG TAC CGG AGG TAA GGG ATC TGG AGG TAA GGG ATC TGA AGT GAA ATT GGT GGA GTC -3'
VHR-54.6	5'- GGC ACT CTA GAG AGG AGA CAG TGA GAG TGG TGC C -3'

### Transformation of *P. pastoris *X33 and selection of recombinants

*P. pastoris *strain X33 was transformed with 10 μg of *Pme I*-linearized pPICZαA-scFv_54.6 _following the Easyselect *Pichia *expression protocol (Invitrogen). In brief, electro-competent cells were prepared from 200 ml of YPD medium inoculated with a fresh culture of *P. pastoris *X33 and incubated at 29°C, shaking at 250 rpm until the OD_600 nm _reached 1.3. The cells then were harvested by centrifugation for five minutes at 1,500 × *g*, washed twice with sterile ice-cold water and once with 10 ml of ice-cold 1 M sorbitol, and then suspended in 0.5 ml of 1 M sorbitol. Electroporation was conducted by mixing 80 μl of this cell suspension with 10 μg of linearized pPICZαA-scFv_54.6 _in a 0.2-cm cuvette. The cells were pulsed with 1.5 kV at a resistance setting of 129 ohms using a BTX ECM 630 electroporator. One ml of 1.0 M sorbitol was immediately added to the pulsed cells which then were transferred to a 15 ml tube and incubated for two hours at 29°C. Cells were plated onto YPD agar supplemented with 1.0 M sorbitol and containing 100 μg/ml of zeocin. Colonies were screened for multi-copy recombinants by patching onto YPD agar plates containing concentrations of zeocin ranging from 100 to 2,000 μg/ml. Recombinants selected on 2,000 μg/ml zeocin were screened for Met^+ ^phenotype by streaking onto minimal methanol agar medium and inserts were confirmed by PCR.

### scFv expression and purification

scFv_54.6 _expression was conducted by growing recombinants in 50 ml buffered complex glycerol medium (BGMY, pH 6.0) for 18 hrs at 29°C. Cells cultured to log phase were harvested by centrifugation at 1,500 × *g*, diluted to an OD_600 nm _of 1.0 in buffered complex methanol medium (BMMY, pH 6.0) to induce expression, and then incubated for 72 hrs at 25°C in a shaking incubator at 250 rpm. At each 24 hour time point, 100% methanol was added to a final concentration of 1.0%. Casamino acids were added to BMMY to a final concentration of 1.0% to reduce proteolysis. One ml samples were taken at selected time points and supernatants were analyzed for scFv_54.6 _expression by SDS-PAGE and immunoblotting using anti-c-myc (Clontech) as the primary antibody.

The pre-cleared supernatant from induced cultures was precipitated by addition of ammonium sulfate to 45% saturation and incubated overnight at 4°C. Proteins were harvested by centrifugation for 25 minutes at 6,700 × *g *and then dialyzed against sodium phosphate saline buffer (50 mM monobasic sodium phosphate, 150 mM NaCl, pH 7.4). The dialysate was diluted with an equal volume of Ni-NTA buffer (50 mM monobasic sodium phosphate, 300 mM NaCl, pH 8.0) containing 25 mM imidazole and then incubated with Ni-NTA beads for 20 minutes at 4°C with end-over-end rotation. Beads were collected by centrifugation for two minutes at 800 × *g *and then washed five times with wash buffer (50 mM monobasic sodium phosphate, 300 mM NaCl, 20 mM imidazole, pH 8.0). His-tagged proteins were eluted with Ni-NTA buffer containing 250 mM imidazole, dialyzed against sodium phosphate buffer, pH 6.5, and concentrated by ultrafiltration through a Centricon Plus-20 column (Millipore).

### Hemagglutination inhibition assay (HI)

The HI assay was performed as described previously [20]. In brief, 50 μl serial dilutions of purified scFv_54.6 _or mAb 54.6 in sterile PBS-H (0.01 M sodium phosphate, 0.15 M sodium chloride, pH 5.5) were added to the wells of a vinyl flexible V-bottom 96-well plate (Costar), followed by addition of 50 μl of a 0.5 % suspension of type O red blood cells. Ten μl of VLPs (5 ng/ml in PBS-H) or PBS-H were added to the appropriate wells indicated in the figure legend. The plate was gently agitated and incubated for two hours at 4°C.

### Construction of CHO-FTB_KE _cells and VLP blocking assays

A stable Chinese Hamster Ovary (CHO) cell line that expresses α 1,2-fucosyltransferase (α 1,2-FT) was constructed as previously described [31]. The rat FTB gene encoding α 1,2-FT was amplified by RT-PCR from total RNA isolated from rat colon using oligonucleotides FTB-F (5' CAA*GGATCC*ATGGCCAGCGCCCAGGTT-3') and FTB-R (5' CAC*CTCGAG*TTAGTGCTTAAGGAGTGGGGAC-3'). The PCR amplicon was cloned in *BamHI *and *XhoI *sites of pcDNA3.1/Myc-His(+)A vector (Clontech). CHO-K1 cells were transfected with the FTB cDNA and stably maintained in RPMI containing 0.4 mg/ml G418. Expression of α 1,2-FT was confirmed by binding of FITC-conjugated UEA-lectin as previously described [31].

The ability of scFv_54.6 _to block binding of VLPs to cells was measured by flow cytometry using R-Phycoerythrin (PE)-conjugated anti-mouse IgG (Jackson Immunoresearch Laboratories). Binding assays were performed as described previously [31]. Briefly, 990 ng of VLPs diluted in PBS (pH 6.7) containing 0.1% gelatin were incubated for two hours at 4°C in the presence or in the absence of scFv_54.6 _(5 μg/ml) or control IgG Fab fragment (Jackson Immunoresearch Labs). The reactions were mixed with 2 × 10^5 ^CHO-FTB_KE _cells and incubated for another two hours. Cells were washed and VLP binding was detected by incubation for one hour with anti-rNV mAb 72.1 [20] followed by a 45 minute incubation with PE-conjugated goat anti-mouse antibody at a final concentration of 2.0 μg/ml. MAb 72.1 recognizes rNV particles but is different from mAb 54.6, as their V_L _and V_H _sequences are not identical (unpublished data). Fluorescent cells were counted on a FACSCalibur (Becton Dickinson) and the data were analyzed with CellQuest Pro software (version 5.2.1; BD Biosciences).

## Competing interests

The author(s) declare that they have no competing interests.

## Authors' contributions

KE performed all of the experimental work in this study and assisted in preparation of the manuscript. MEH conceived of the study, participated in study design, jointly prepared the manuscript and is responsible for oversight of the entire project. Publication of the final manuscript is approved by the authors.
